# Investigation of the Defect and Intensity-Dependent Optical Limiting Performance of MnO_2_ Nanoparticle-Filled Polyvinylpyrrolidone Composite Nanofibers

**DOI:** 10.1021/acsomega.3c06572

**Published:** 2023-12-07

**Authors:** Yasemin Pepe, Serife Akkoyun, Nurcan Asci, Eda Cevik, Yusuf Tutel, Ahmet Karatay, Husnu Emrah Unalan, Ayhan Elmali

**Affiliations:** † Department of Engineering Physics, Faculty of Engineering, Ankara University, 06100 Ankara, Türkiye; ‡ Department of Metallurgical and Materials Engineering, Faculty of Engineering and Natural Sciences, Ankara Yildirim Beyazit University, 06010 Ankara, Türkiye; § Central Research Laboratory, Application and Research Center, Ankara Yildirim Beyazit University, 06010 Ankara, Türkiye; ∥ Department of Metallurgical and Materials Engineering, Middle East Technical University (METU), 06800 Ankara, Türkiye; ⊥ Energy Storage Materials and Devices Research Center (ENDAM), Middle East Technical University (METU), 06800 Ankara, Türkiye

## Abstract

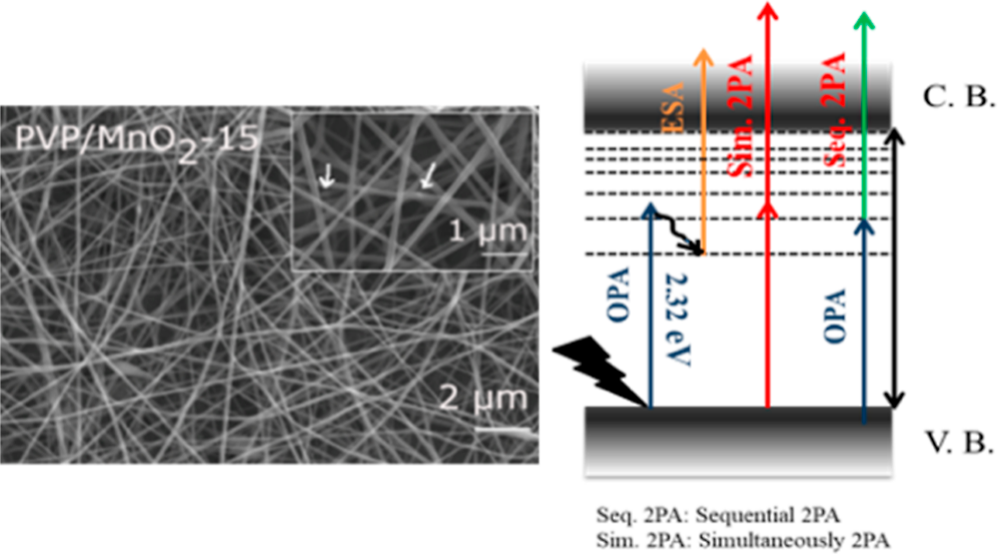

To enhance the optical limiting behavior triggered by nonlinear absorption (NA), wide-band gap MnO_2_ nanoparticles were incorporated into polyvinylpyrrolidone (PVP) polymer nanofibers at various concentrations. SEM images of the composite nanofibers showed that MnO_2_ nanoparticles are well entrapped in the nanofibers. With an increase in MnO_2_ nanofiller concentration, a widened optical band gap energy and an increased Urbach energy were observed. As the concentration of MnO_2_ nanofiller in PVP increased, the NA behavior became more pronounced but weakened with higher input intensity. This behavior was attributed to the filling of the localized defect states by one photon absorption (OPA). The NA mechanisms of the composite nanofibers were examined, considering their band gap energies and localized defect states. Although all of the composite nanofibers had OPA, sequential/simultaneous two photon absorption (TPA), and excited state absorption mechanisms, the higher concentration of the MnO_2_ nanofiller led to stronger NA behavior due to its more defective structure. The highest optical limiting behavior was observed for composite nanofibers with the highest concentration of MnO_2_ nanofiller. The results obtained show that these composite nanofibers with a high linear transmittance and an extended band gap energy can be used in optoelectronic applications that can operate in a wide spectral range. Furthermore, their robust NA behavior, coupled with their promising optical limiting characteristics, positions them as strong contenders for effective optical limiting applications.

## 1 Introduction

Recently, transition metal oxides (TMOs) have garnered substantial attention thanks to their distinct optical, electrical, and chemical properties, alongside their auspicious morphological structures. Moreover, TMOs have also emerged as nonlinear optical materials, owing to their remarkable optical nonlinearity, rapid response times, and robust chemical stability.^1^ Among TMOs, MnO_2_ stands out for its exceptional corrosion resistance, cost-effectiveness, straightforward synthesis methods, and environmentally friendly attributes.^2^ It is also an attractive nonlinear optical material with a wide range of adaptable physical and chemical properties, such as a narrow band gap and a high optical constant.^3−5^ Various synthesis methods of MnO_2,_ such as hydrothermal,^2,6,7^ precipitation,^8^ sol–gel,^9−11^ reduction,^12^ and electrodeposition^13,14^ have been reported in the literature. MnO_2_ exhibits polymorphism, manifesting in a range of phases such as α-, β-, γ-, δ-, λ-, and ε-phases. These variations arise from the distinct configurations of the fundamental [MnO_6_] octahedra, interlinked through the sharing of edges and corners.^6,15−17^ This polymorphism contributes to the emergence of numerous distinctive physical and chemical properties in MnO_2_. Out of all the different phases, α-MnO_2_ stands out with its remarkable characteristics, including a high specific surface area, low scattering, abundant active sites, broad light absorption, excellent electron transfer capability, and exceptional mechanical flexibility.^18^ Despite extensive research conducted on the use of MnO_2_ in applications such as supercapacitors,^19−21^ batteries,^22−24^ electrocatalysts,^25−27^ and sensors^28,29^ there have been relatively few studies focusing on its potential use in optoelectronic and nonlinear optical applications. Kumar et al. investigated the optical nonlinearity of the β-MnO_2_ nanowires decorated with Ag nanoparticles,^30^ and they reported that the Ag-decorated β-MnO_2_ had stronger optical limiting behavior as compared with the neat β-MnO_2_ nanowires. Dhanusha and Sabari Girisun investigated the influence of the morphology of α-MnO_2_ (nanoclusters, nanorods, and nanowires) on optical nonlinearity,^31^ and they reported that the α-MnO_2_ nanowires with an extended interconnected network and a larger aspect ratio exhibit a higher two-photon absorption coefficient and a lower onset optical limiting threshold.

Polyvinylpyrrolidone (PVP) is a nontoxic polymer that is soluble in solvents such as ethanol and methanol, and especially in water. Consequently, it finds extensive application in industries such as cosmetics,^32^ pharmaceuticals,^33^ and the food industry.^34^ Polymer nanocomposites present distinctive thermal, electrical, and optical properties, synergistically combined with the inherent benefits of polymers, including transparency, processability, and flexibility.^35,36^ Besides, nanocomposites have been extensively investigated for their potential applications such as optical limiting, optical switching, and light-controlled phase due to the combination of their unique properties.^37−44^ Deena et al. investigated the nonlinear absorption and optical limiting properties of (Mn and W) oxides decorated with nitrogen-doped reduced graphene oxide nanocomposites.^45^ They reported that the MnO_2_-decorated NrGO nanocomposites, as compared to their pure analogs and derivatives of graphene had a higher two-photon absorption coefficient and the lowest optical limiting threshold. Besides, recently we investigated the optical limiting behavior of bare and doped (Cu and Co) poly(methyl methacrylate)/α-MnO_2_ nanocomposite films^46^ and it was reported that Co-doped MnO_2_ nanocomposite films had stronger optical limiting behavior. On the other hand, to the best of our knowledge, there is no study examining the optical limiting and nonlinear absorption (NA) properties of MnO_2_ nanoparticle-filled PVP composite nanofibers. Nanofibers filled with nanoparticles are a good method to observe the nonlinear absorption of nanoparticles; otherwise, sensitive measurements cannot be made suspended in the solvent. In addition, the different energy band gaps of the nanoparticle and the polymer widen the spectrum in which nonlinear absorption is observed. Thus, NA and optical limiting performance can be increased with different adsorption mechanisms, such as OPA and TPA. Owing to their small dimensions, nanoparticles have an extremely high surface-to-volume ratio. This property increases the light–matter interaction compared to bulk materials. Besides, nanofibers are materials with a high aspect ratio (length to diameter ratio), and this property supports increased light-matter interaction like in nanoparticles. The combined system (nanofibers filled with nanoparticles) should have a higher light-matter interaction than each material. The nanofibrous structure of electrospun mats allows high light-matter interactions, which greatly affects the nonlinear optical character of the materials. For this reason, the first aim of this work is to effectively produce MnO_2_-filled PVP composite nanofibers. The second aim is to reveal the effect of NA mechanisms on the optical limiting behavior of the PVP/MnO_2_ composite nanofibers. To provide a higher contribution of localized defect states to NA mechanisms, the excitation wavelength was chosen at 532 nm for open-aperture Z-scan experiments, which were conducted at increasing input intensities.

## 2 Materials and Methods

### 2.1 Materials

Potassium permanganate (KMnO_4_) and ethylene glycol (HOCH_2_CH_2_OH) were obtained from Sigma-Aldrich and used as received without purification. Polyvinylpyrrolidone (PVP—K85–95) with a molecular weight of 1300 kg/mol was purchased from ACROS Organics. Ethanol with a purity of 96% was purchased from Aytaş (Türkiye).

### 2.2 Synthesis of MnO_2_ Nanoparticles and the Production of PVP/MnO_2_ Composite Nanofibers

The synthesis of undoped α-MnO_2_ powders followed a slightly modified reduction method, as previously described by Dong et al.^12^ A precursor solution of α-MnO_2_ was prepared by dissolving 0.045 M KMnO_4_ in 200 mL of deionized (DI) water, followed by the gradual addition of 10 mL of ethylene glycol (EG). The resulting solution was stirred for 3 h and then subjected to multiple washing steps using DI water and ethanol. The obtained solids were subsequently dried at 80 °C overnight. Finally, the powders were heat-treated in air at 450 °C for 3 h.

**Figure 1:**
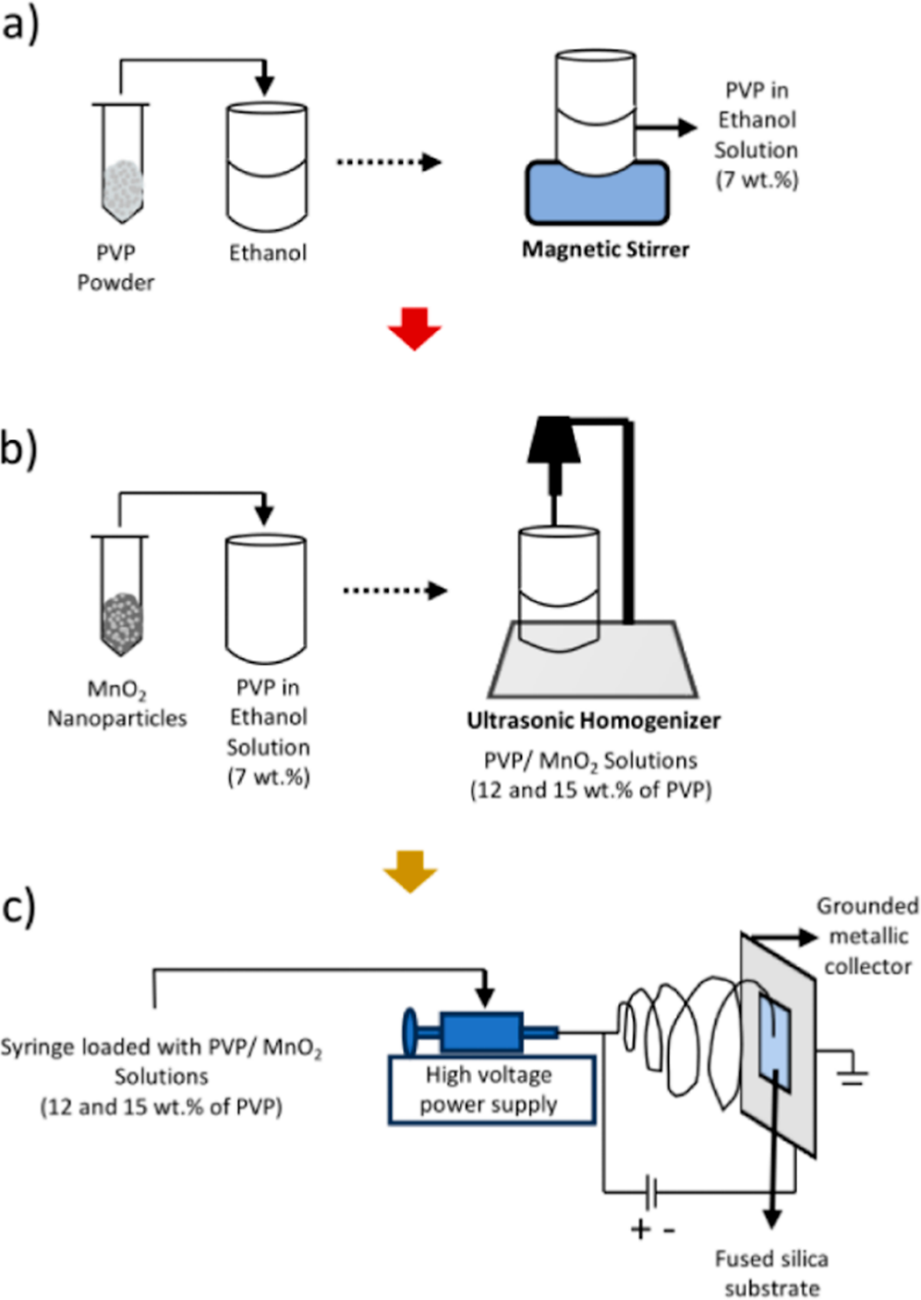
Schematic representation of (a) preparation of PVP in ethanol solutions, (b) preparation of PVP/MnO_2_ solutions, and (c) electrospinning of PVP and PVP/MnO_2_ composite nanofibers.

PVP in ethanol solutions of 7 wt % were prepared under constant stirring until complete dissolution of the polymer. Then, PVP/MnO_2_ solutions with various MnO_2_ nanoparticle contents (12 and 15 wt % of PVP) were prepared by adding to the polymer solutions the appropriate amounts of nanoparticles, which were dispersed using an ultrasonic homogenizer (BANDELIN GM 2200) for 2 h (Figure 1a,b). In order to preserve the homogeneity of the PVP/MnO_2_ solutions, electrospinning was carried out immediately after ultrasonic homogenization. The obtained composite nanofibers were labeled as PVP/MnO_2_-12 and PVP/MnO_2_-15 for 12 and 15 wt % of MnO_2_ nanofiller contents, respectively. PVP and PVP/MnO_2_ composite nanofibers (PVP/MnO_2_) were electrospun with a feed rate of 1.25 mL/h, a high voltage of 17.5 kV, and a tip-to-collector distance of 15 cm. All of the samples were produced on fused silica substrates in order to perform optical characterizations (Figure 1c).

### 2.3 Characterization

The MnO_2_ nanopowders synthesized were morphologically characterized by using scanning electron microscopy (SEM) with an energy dispersive X-ray (EDX) analyzer, specifically the FEI Nova Nano FEG-SEM, at 20 kV. Prior to SEM analysis, the nanopowders were coated with a thin layer of gold (Au) to improve SEM precision. The crystal structure of the powders was determined through X-ray diffraction (XRD) analysis performed using a Rigaku D/Max-2000 diffractometer with Cu Kα radiation at 40 kV and a wavelength of 0.154 nm. The XRD analysis was conducted between 10 and 80° at a scanning rate of 1° per minute. The average particle size of the nanoparticles that were dispersed in water was determined by photon correlation spectroscopy in water using a Malvern Nano ZS (Malvern Instruments, UK). The morphologies of PVP and PVP/MnO_2_ composite nanofibers were characterized with a HITACHI SU5000 field emission scanning electron microscope (SEM) equipped with an Oxford X-MaxN 80 EDS detector, which was used in obtaining energy dispersive X-ray spectroscopy (EDS) maps. For a better contrast, aluminum tape was used for mounting the samples in SEM-EDS observations. The average diameters were determined using 100 nanofibers with the aid of ImageJ software (NIH—USA). A Woollam M2000 V spectroscopic ellipsometer was used to determine the thickness of the electrospun nanofiber mats on fused silica substrates at three angles of incidence (65, 70, and 75°). The measurements showed that the average sample thickness was 700 nm. A Shimadzu UV-1800 model UV–vis spectrophotometer was used to reveal the linear absorption behavior of the PVP/MnO_2_ composite nanofibers. Photoluminescence measurements were performed under excitation wavelengths of 400 and 300 nm using a PerkinElmer LS55 spectrophotometer. A Q-switched Nd:YAG (Quantel Birillant) laser (10 Hz repetition rate and 4 ns pulse duration) was used at an excitation wavelength of 532 nm in OA Z-scan experiments to reveal the effective nonlinear optical behaviors of the nanofibers.

## 3 Results and Discussion

### 3.1 Structural and Morphological Analysis of MnO_2_ Nanoparticles and PVP/MnO_2_ Composite Nanofibers

**Figure 2:**
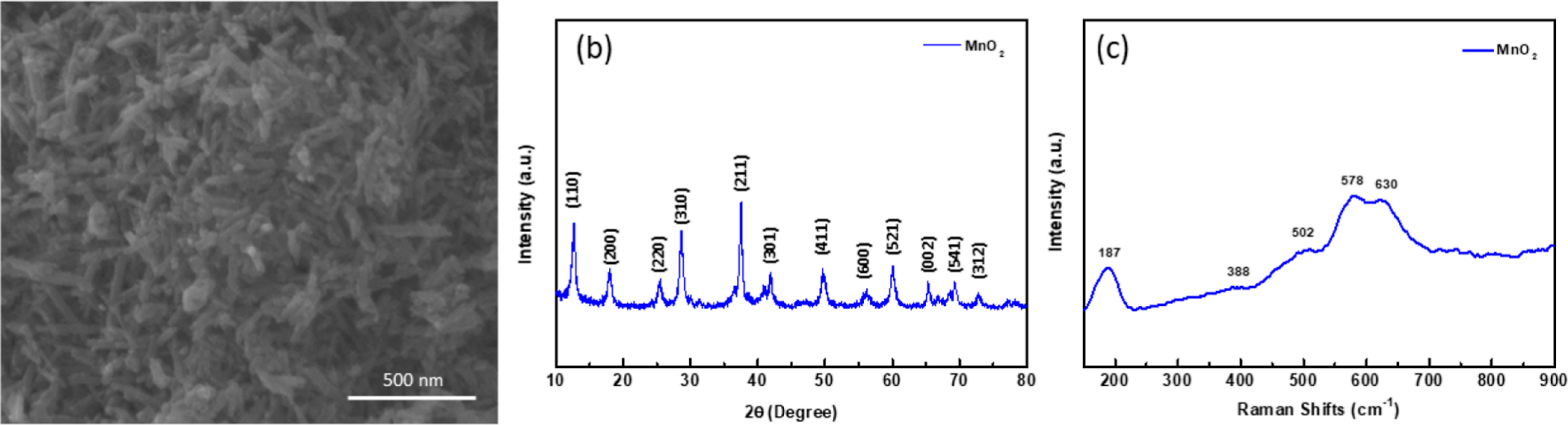
(a) SEM image, (b) XRD pattern, and (c) Raman spectra of the synthesized α-MnO_2_ nanopowder.

The morphological investigation of α-MnO_2_ nanoparticles was conducted using the SEM analysis result given in Figure 2a. The SEM image primarily illustrates the presence of nanorods, along with some aggregated nanoparticles. Similar morphologies were also observed in the literature on bare α-MnO_2_ nanoparticles synthesized by the reduction method.^47,48^ All nanoparticles were dispersed in water to determine their average particle size by photon correlation spectroscopy. The *d* (0.5) values were found to be around 64 nm. The XRD patterns of the α-MnO_2_ powders exhibit a precise match with the JCPDS card no. 44-0141,^49−51^ as shown in Figure 2b. The diffraction peaks can be assigned to the (110), (200), (220), (310), (211), (301), (411), (600), (521), (002), (541), and (312) planes, indicating the tetragonal structure of α-MnO_2_ with a cryptomelane-type network.^52,53^ No discernible peaks corresponding to other MnO_2_ phases, such as γ- and β-MnO_2_, were observed in the XRD pattern. This observation proved the high purity of the synthesized products. Furthermore, sharp reflection peaks signified the crystalline nature of the obtained materials. Raman spectroscopy was employed to further characterize the structural properties of α-MnO_2_ nanoparticles. The sample exhibited five distinct Raman peaks, as shown in Figure 2c. The first peak observed at 187 cm^–1^ was attributed to external vibrations.^49,54,55^ The peaks observed at 388 and 502 cm^–1^ were assigned to the deformation modes of Mn–O–Mn bonds,^56,57^ whereas the last two peaks located around 578 and 630 cm^–1^ were ascribed to the Mn–O chain tensile vibration and the symmetric tensile vibration of the [MnO_6_] group, respectively.^49,55,58,59^ Additionally, the presence of less distinct peaks at 388 and 502 cm^–1^ indicates the phonon density of the state rather than the region of center phonons allowed by Raman. This suggests that phonons are restricted due to crystal defects and localized lattice distortions in α-MnO_2_ nanopowders.^60,61^ On the other hand, some broadening and shifting of Raman active modes compared to similar studies in the literature is due to the increase in the number of oxygen vacancies formed by the displacement of oxide ions from their normal cages.^61^ These two distinct Raman peaks observed around 578 and 630 cm^–1^ from the Mn–O stretching range suggested a well-developed tetragonal structure that is composed of (2 × 2) tunnels.^49,58,59^ The Raman spectra findings provided successful support for the results obtained from the XRD analysis. Considering the structural analyses, it becomes apparent that the synthesized α-MnO_2_ nanopowder exhibits a cryptomelane-type phase.

**Figure 3:**
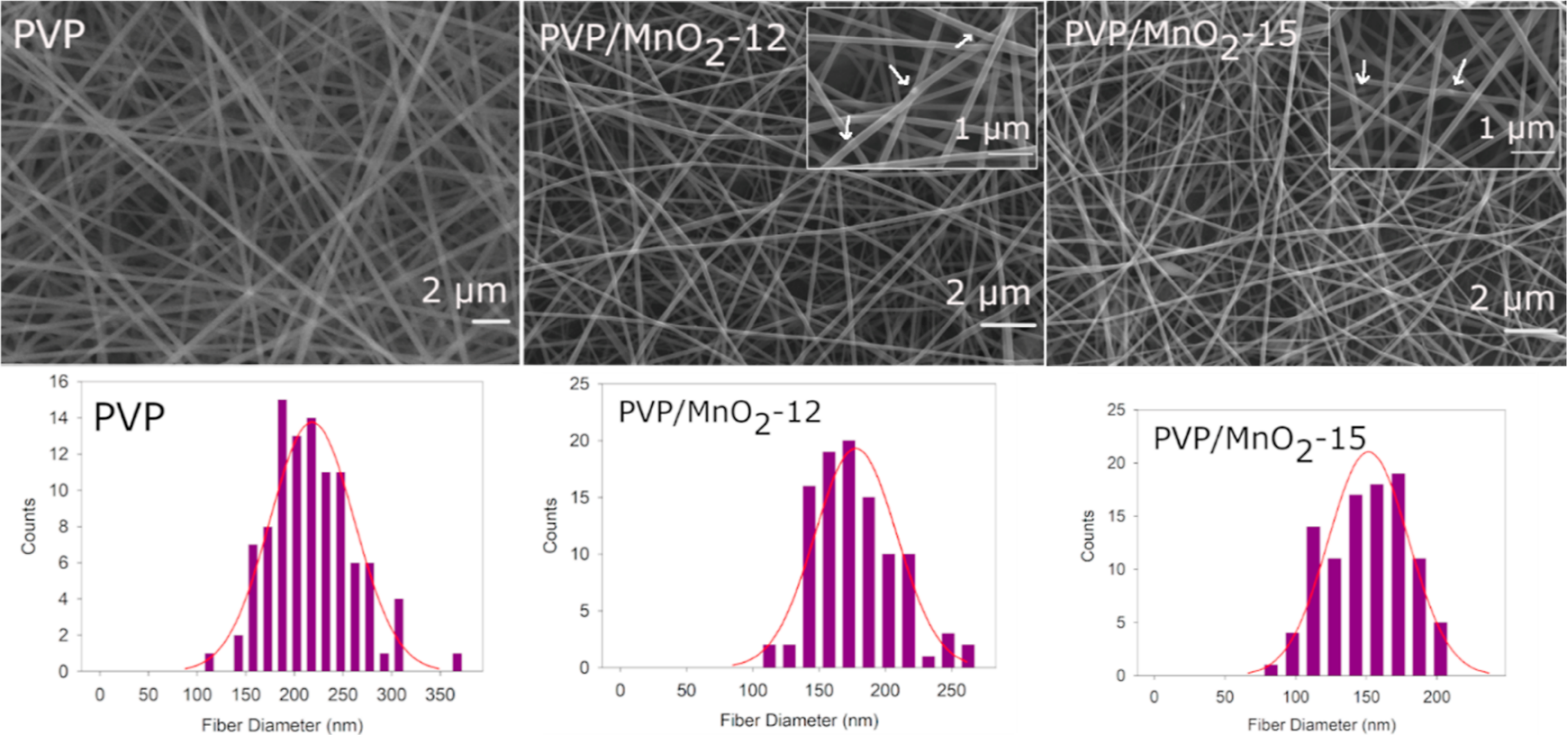
SEM micrographs and diameter distributions of PVP and PVP/MnO_2_ composite nanofibers. Close-up pictures are given as the inset for PVP/MnO_2_ composite nanofibers.

**Figure 4:**
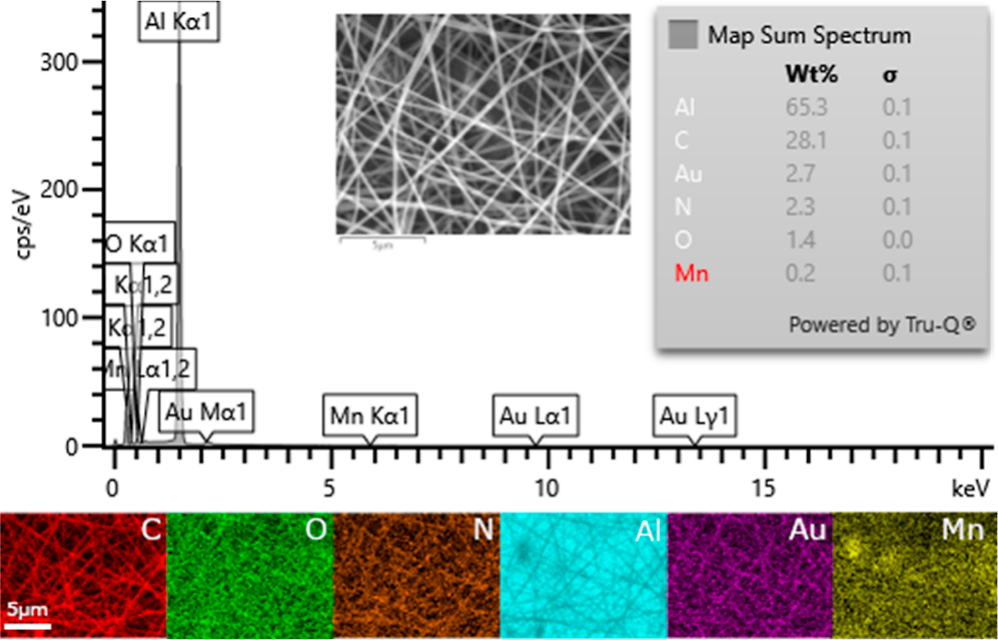
EDS spectrum and corresponding elemental maps and compositions of the PVP/MnO_2_ composite nanofibers. The analysis area is given as an inset.

The SEM micrographs and diameter distributions of PVP and PVP/MnO_2_ composite nanofibers are provided in Figure 3. The average diameters are listed in Table 1. The results show that cylindrical and homogeneous nanofibers were produced in all cases. Although the presence of nanoparticles decreased the diameter of nanofibers, it did not affect their morphology. Moreover, the diameter of the nanofibers also decreased with the filler content. Similar results were observed in the literature.^62^ Nanoparticle aggregates are more discernible and are indicated by white arrows in the micrographs. SEM images also show that MnO_2_ nanoparticles are well incorporated into the nanofibers. The composition of the nanofibers was also confirmed by EDS measurements. The EDS spectrum and corresponding elemental maps of PVP/MnO_2_ composite nanofibers are provided in Figure 4. As PVP is mainly composed of carbon (C), oxygen (O), and nitrogen (N), these elements are particularly located on the nanofibers. Manganese (Mn) is localized on MnO_2_ aggregates, but it is also present on the whole sample as the distributed MnO_2_ nanoparticles were too small for SEM observation. The samples were sputter-coated with gold (Au), which also appeared in the elemental map. Besides, the presence of aluminum (Al) was attributed to the aluminum tape.

**Table 1:** Average Diameters of PVP and PVP/MnO_2_ Composite Nanofibers

nanofibers	average diameters (nm)
PVP	218 ± 43.4
PVP/MnO2-12	178 ± 30.9
PVP/MnO2-15	152 ± 28.4

### 3.2 Linear Absorption Analysis

**Figure 5:**
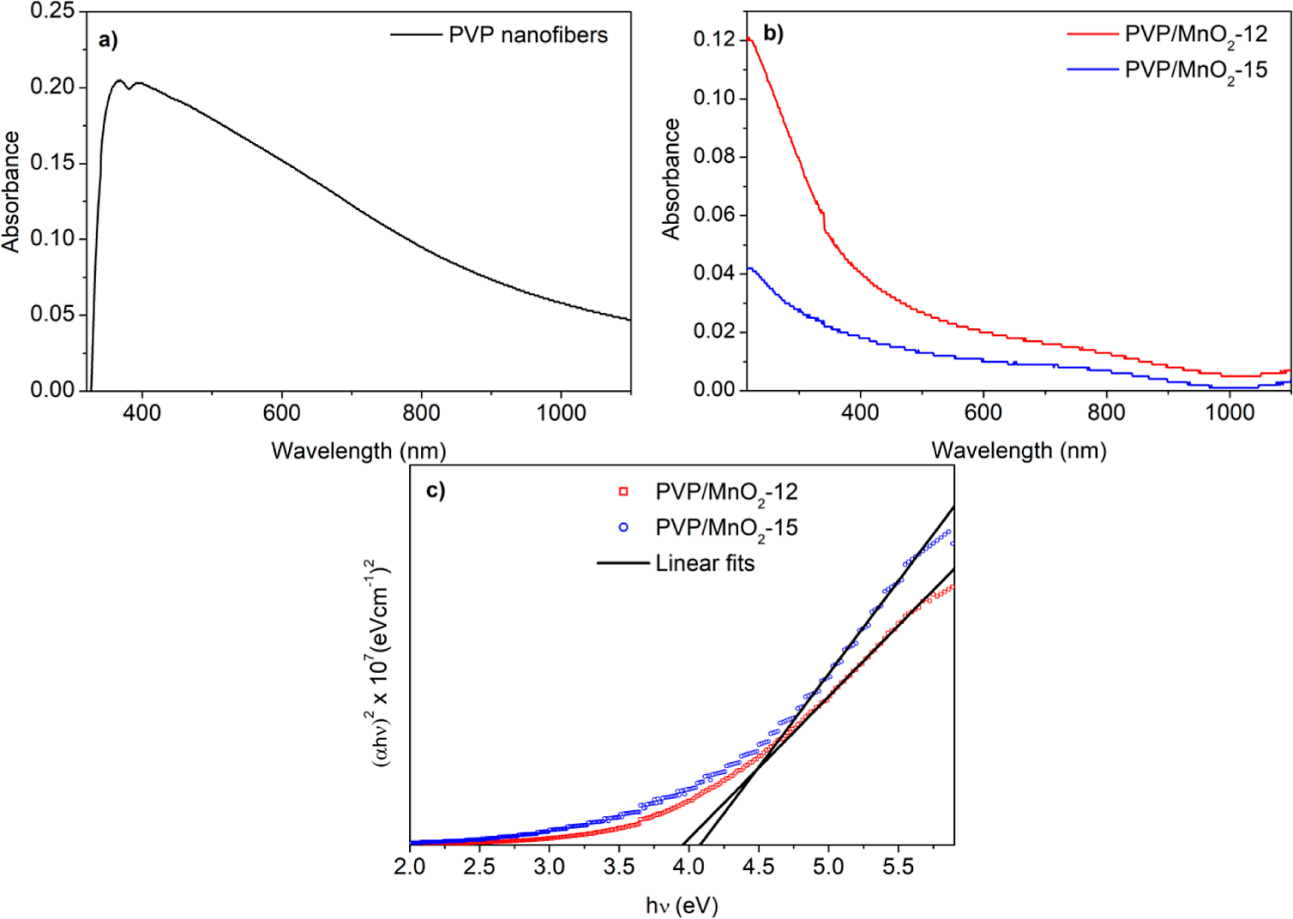
Absorbance spectra of (a) pure PVP nanofibers and (b) PVP/MnO_2_ composite nanofibers. (c) (α*h*ν)^2^ vs *h*ν graphs (Tauc plots) of PVP/MnO_2_ composite nanofibers.

The linear optical absorption spectra of the PVP/MnO_2_ composite nanofibers are shown in Figure 5a. The absorption spectrum of the nanofibers shows a maximum in the blue region at 230 nm, and it slightly decreases and expands to the near-infrared region. As seen from this figure, the absorption capability of the composite nanofibers decreased with increasing MnO_2_ nanofiller content in PVP. It is well known that the nanofiber diameter strongly affects the light scattering behavior of nanofiber mats.^63,64^ Decreasing the diameter of PVP/MnO_2_ composite nanofibers caused the absorbance to decrease. Understanding the band gap and Urbach energies is of paramount significance as they elucidate the NA mechanism within the studied composite nanofibers. Therefore, their band gap energies were found using the following expression^65^ and the absorption spectra of the materials.
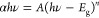
1where *n* = 1/2 and *n* = 2 for direct and indirect transitions taking place between valence and conduction bands, respectively, *E*_g_ is the band gap energy, *A* is a constant, *h*ν is the photon energy, and α is the absorption coefficient. The band gap energy was found to be 3.95 eV and increased to 4.07 eV with increasing MnO_2_ nanofiller content in PVP.

**Figure 6:**
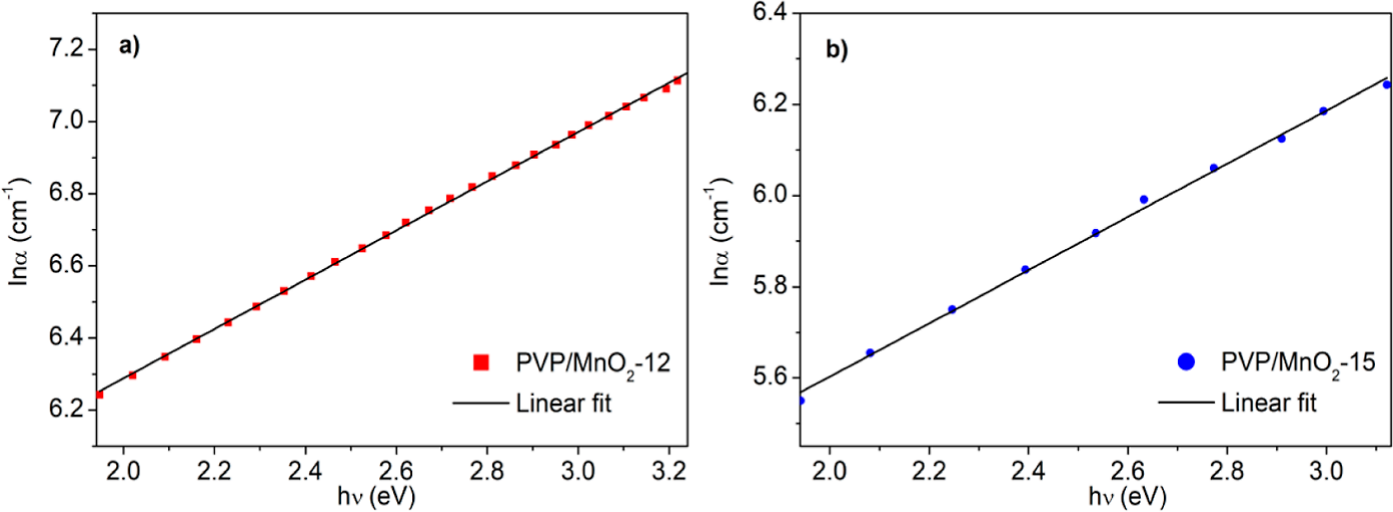
*ln* α vs *hν* plot of (a) PVP/MnO_2_-12 and (b) PVP/MnO_2_-15 composite nanofibers.

The slightly increased absorption band edges in Figure 5a indicate the defect states inside the band gap. To find out the defect state density of the composite nanofibers, their Urbach energies were determined using the following expressions^66^ and absorption data for the materials.
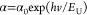
2where *E*_U_ is the Urbach energy, α_0_ is a constant, and α is the absorption coefficient. In the ln*α* versus *hν* graph, the inverse slope of the linear region gives the Urbach energy of the material. ln α versus *hν* plots of the composite nanofibers are provided in Figure 6. Urbach energy values were found to be 1.46 and 1.71 eV for PVP/MnO_2_-12 and PVP/MnO_2_-15 composite nanofibers, respectively. The observed increase in Urbach energy in PVP/MnO_2_-12 compared to PVP/MnO_2_-15 composite nanofibers can be attributed to the increase of defects. In other words, the increase in Urbach energy with increasing MnO_2_ nanofiller content within PVP indicates a concurrent increase in defect states within the band gap.

**Figure 7:**
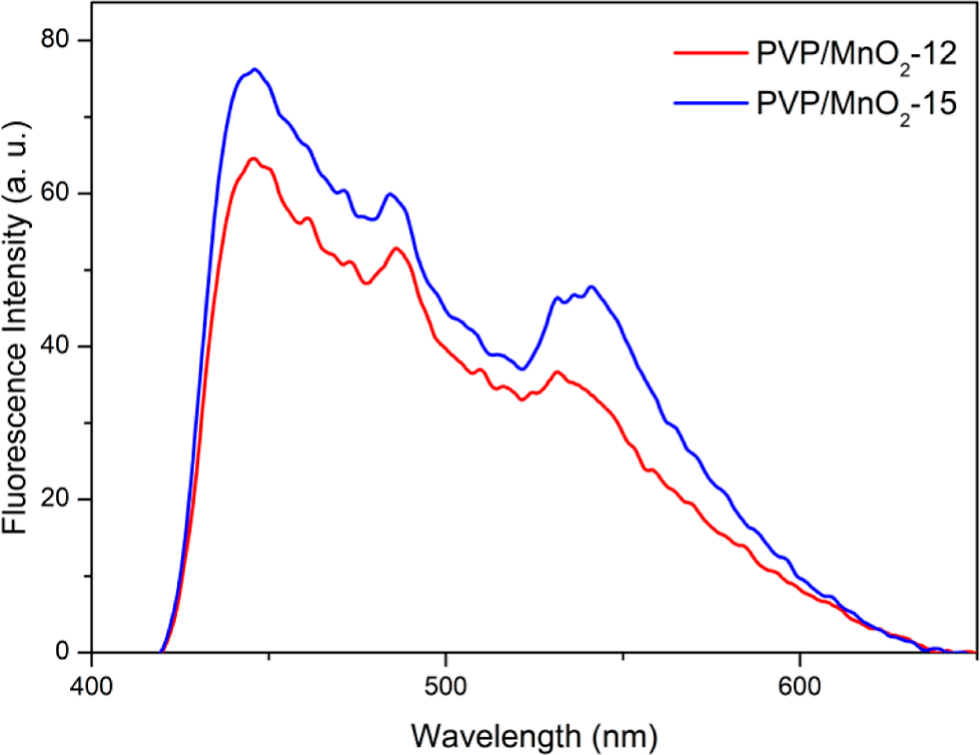
Photoluminescence spectra of the PVP/MnO_2_ composite nanofibers.

The photoluminescence spectra of the pure PVP nanofibers and PVP/MnO_2_ composite nanofibers are shown in Figure 7 and were obtained under excitation wavelengths of 400 and 300 nm, respectively. Both composite nanofibers had emission signals at 446, 484, and 540 nm. These emitted wavelengths correspond to the absorption band edge. Therefore, these emission signals were due to transitions between the defect states and the valence band. Additionally, it was observed that the fluorescence intensity originating from these states exhibited a rise in response to an increase in the MnO_2_ nanofiller content within the PVP matrix.

### 3.3 Nonlinear Absorption Analysis

**Figure 8:**
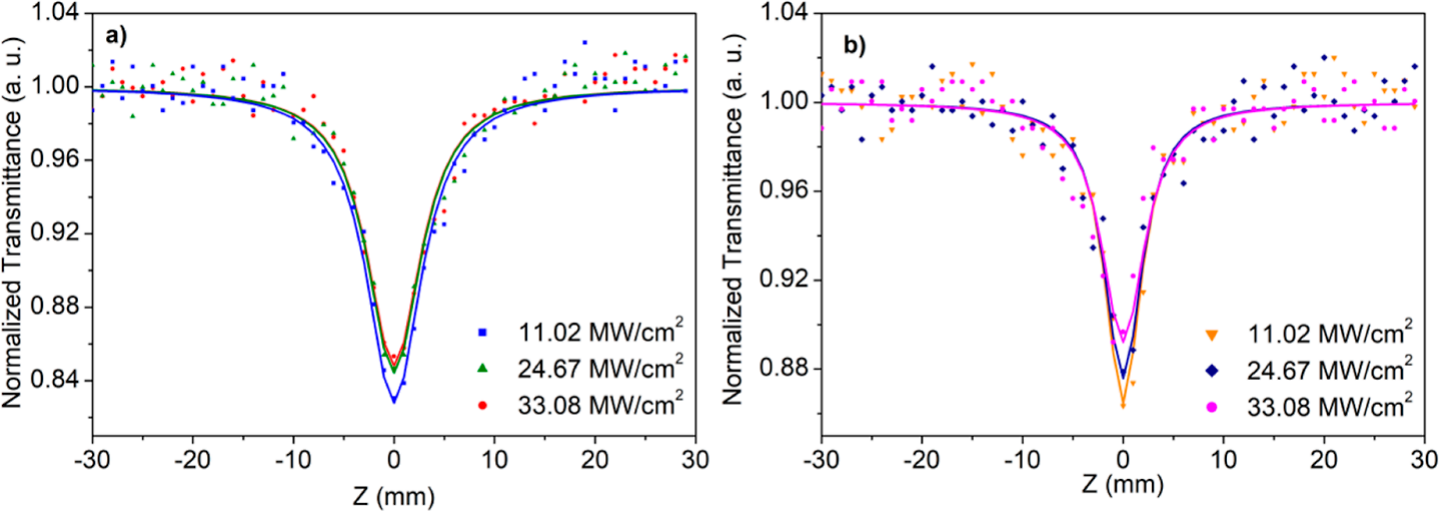
Open aperture Z-scan curves with theoretical fits of (a) PVP/MnO_2_-12 and (b) PVP/MnO_2_-15 composite nanofibers at various input intensities.

The OA Z-scan experiments of the PVP/MnO_2_ composite nanofibers were conducted at 532 nm (corresponding to 2.32 eV) considering the band gap energy values. The OA Z-scan curves with their theoretical fits are listed in Figure 8. According to the width of the experimental data, it can be said that the one photon absorption (OPA) contribution to NA was much stronger for a filler content of 12% wt. compared to 15%. This result was also supported by the higher linear absorption of PVP/MnO_2_-12 as compared to that of PVP/MnO_2_-15 composite nanofibers (see Figure 5). Besides, an increasing normalized transmittance was observed with increasing input intensity for both composite nanofibers. This means that the defect states corresponding to the excitation wavelength (2.32 eV) were filled with OPA from the valence band.

To reveal the nonlinear absorption parameters, such as nonlinear absorption coefficients (β_eff_) and saturation intensity threshold (*I*_SAT_), a comprehensive theoretical fit model (eq 3) was used. With this theoretical fit, all possible absorptions that may contribute to NA, such as valence band to conduction band, valence band to defect states, defect states to conduction band, and free carrier absorptions, were considered. In this model, the first term represents the OPA and its saturation, the second term represents the two-photon absorption (TPA) and its saturation, and the third term represents the free carrier absorption (FCA) and its saturation.
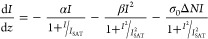
3Where Δ*N*(*I*) is the generated photocarrier density given as
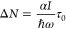
4

The following expression can be obtained by substituting eq 4 in eq 3.
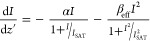
5
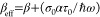
6where α is the linear absorption coefficient, ℏω is the photon energy, β is the TPA coefficient, β_eff_ is the effective NA coefficient, τ_0_ is the pulse duration, and σ_0_ is the FCA cross section. Fitting details can be found in the literature.^67^ The laser pulse energies were chosen as 0.7, 1.5, and 2 μJ for PVP/MnO_2_-12 and 0.5, 1.1, and 1.5 for PVP/MnO_2_-15 composite nanofibers. The ω_0_ value was obtained from the fitting of the experimental data. The values of ω_0_ were obtained to be 22 and 19 μm, and *z*_0_ values were found to be 0.028 and 0.019 cm for the PVP/MnO_2_-12 and PVP/MnO_2_-15 composite nanofibers, respectively. The obtained fitting results are listed and are provided in Table 2. According to these results, both composite nanofibers have stronger NA at lower input intensity (11.02 MW/cm^2^), and their NA behavior becomes weak with increasing input intensity. This result indicates the filling of the defect states by OPA. On the other hand, these filled defect states, which are signs of saturable absorption, did not completely eliminate NA due to the highly defective structure of the composite nanofibers at the used input intensity. They only caused a decrease in NA. High NA coefficient values were obtained as 1.02 × 10^–5^ and 1.18 × 10^–5^ m/W at 11.02 MW/cm^2^ for PVP/MnO_2_-12 and PVP/MnO_2_-15 composite nanofibers, respectively. The higher β_eff_ values of PVP/MnO_2_-15 compared to PVP/MnO_2_-12 nanofibers were attributed to their more defective structure. The *I*_SAT_ values of the composite nanofibers decreased with increasing input intensity, similarly to the β_eff_ values. Both composite nanofibers have OPA, TPA, and ESA mechanisms that contribute to NA. On the other hand, the more defective structure of PVP/MnO_2_-15 as compared to PVP/MnO_2_-12 composite nanofibers led to stronger NA behavior for PVP/MnO_2_-15 nanofibers. The effect of the defect states on the NA has been reported in our previous studies.^68−71^ The NA coefficient values of reported studies are listed in Table 3 to compare with the present report. The obtained NA coefficients of the PVP/MnO_2_ composite nanofibers were higher than the values of reported studies in Table 3 excited under similar experimental conditions. The NA parameters are affected by the material’s preparation method, doping, morphology, defect levels and their distribution, and annealing. However, unlike the studies listed in Table 3, in this study, the contribution of defect states to NA was taken into account and added to the fit equation (eq 3). The obtained NA coefficient was obtained by taking into account the contributions of the OPA (absorption from the valence band to defect states), TPA (sequential/simultaneously), and FCA to NA. This contributed to achieving greater NA coefficients than in the studies listed in Table 3.

**Table 2:** Nonlinear Absorption Coefficients (β_eff_), Saturation Intensity Threshold (*I*_SAT_), and Optical Limiting Threshold Values of the PVP/MnO_2_ Composite Nanofibers

	PVP/MnO_2_-12			PVP/MnO_2_-15		
*I*_0_ (MW/cm^2^)	*I*_SAT_ (W/m^2^)	βeff × 10–6 (m/W)	onset op.lim.thres. × 10^–5^ (J/cm^2^)	*I*_SAT_ (W/m^2^)	βeff × 10–6 (m/W)	onset op.lim.thres. × 10^–5^ (J/cm^2^)
11.02	7.88 × 10^11^	10.2 ± 0.46	3.81 ± 0.17	5.31 × 10^12^	11.8 ± 0.53	2.94 ± 0.13
24.67	7.57 × 10^11^	6.89 ± 0.31		3.10 × 10^12^	5.85 ± 0.26	
33.08	4.25 × 10^11^	5.97 ± 0.27		2.34 × 10^12^	3.67 ± 0.16	

**Table 3:** Comparison of NA Coefficients of Reported Nonlinear Optical Materials

samples	β (m/W)	ref
Ag/β-MnO2 (5 ns, 532 nm)	8.32 × 10^–^^10^	(30)
M5 nanowire (9 ns, 532 nm)	(2.01 ± 0.100) × 10^–^^10^	(31)
NGO/MnO2 (9 ns, 5232 nm)	8.92 × 10^–^^11^	(45)
CAN/PVP/Au nanofibers (5 ns, 532 nm)	27.1 × 10^–^^11^	(72)

**Figure 9:**
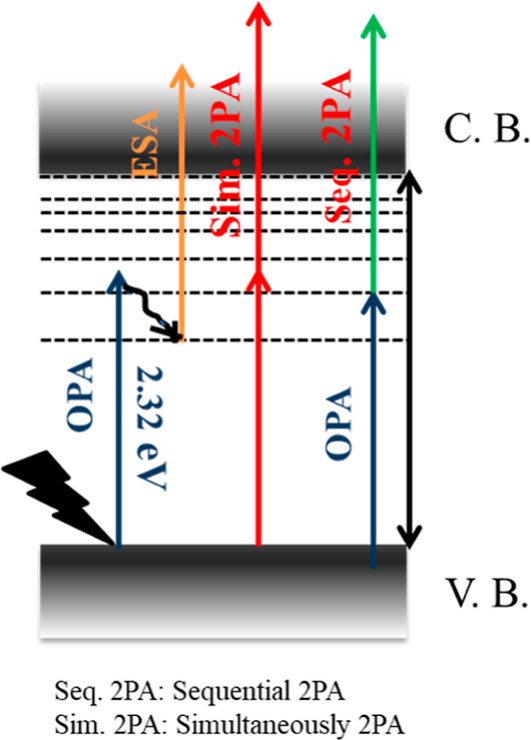
Schematic of the proposed NA mechanisms for PVP/MnO_2_ composite nanofibers.

The optical-limiting behavior of a material is closely related to its NA mechanisms. Considering the band gap and defect states of the nanofibers considered in this study, the OPA from the valence band was sufficient to excite an electron from this state to the defect states of both composite nanofibers. Some of these electrons could lose their energy and make transitions to the valence band. On the other hand, some of these electrons in defect states could be excited to the conduction band by the absorption of another photon, which is known as excited state absorption (ESA). Additionally, an electron could be excited from the valence band to the conduction band by sequential TPA, and a weak contribution could come to NA from FCA in the conduction band. Although both composite nanofibers have the same NA mechanisms, the contribution from these mechanisms to NA at the same input intensity was higher for the PVP/MnO_2_-15 sample due to its higher defect states. A schematic of the proposed NA mechanisms for the nanocomposite mats is provided in Figure 9.

**Figure 10:**
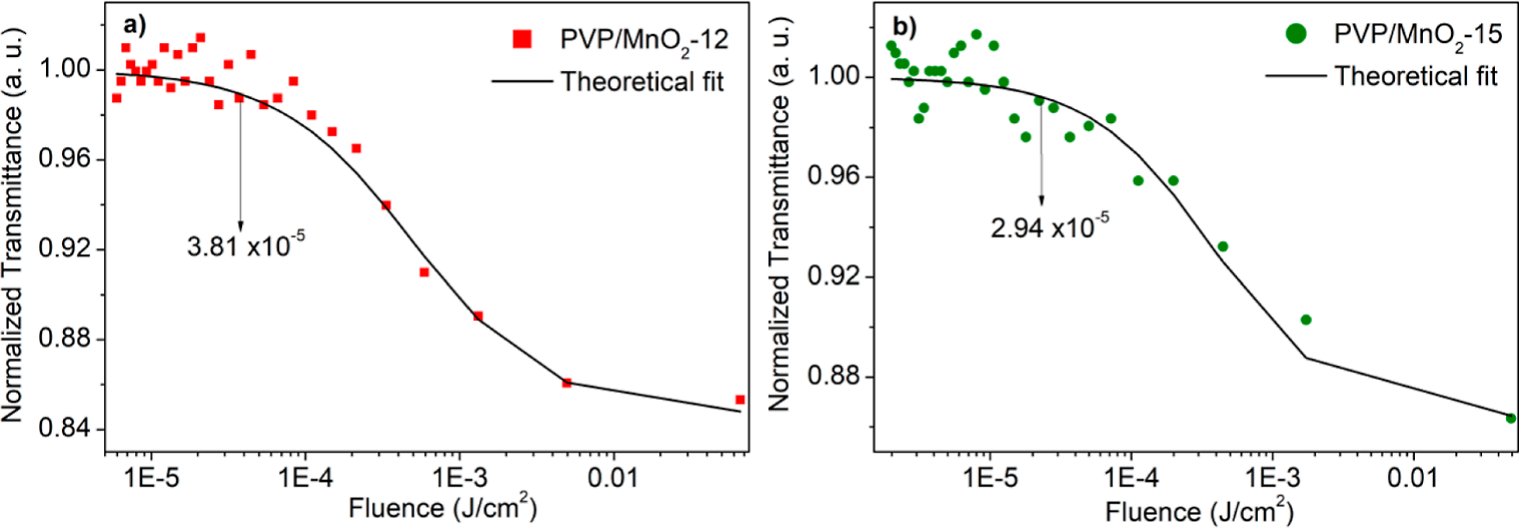
Optical limiting curves of (a) PVP/MnO_2_-12 and (b) PVP/MnO_2_-15 composite nanofibers at an input intensity of 11.02 MW/cm^2^.

High-power laser technology has a detrimental effect on society since it can lead to unintentional exposure to strong laser pulses that can permanently harm optical detectors and eyes. This has inspired researchers to look into and develop novel materials with strong and high linear transmittance, broad-band passive optical limiting, rapid response rates, and high thresholds for laser damage.^73−75^ An ideal optical limiter protects sensitive equipment by limiting transmitted light above the optical limiting thresholds. Stronger NA [TPA, excited state absorption (ESA), and FCA], nonlinear scattering, and nonlinear refraction features cause stronger optical limiting of the materials under high input intensity.^76^ The optical limiting curves of the composite nanofibers at 11.02 MW/cm^2^ input intensity are provided in Figure 10. The onset optical limiting thresholds were determined as 3.81 × 10^–5^ and 2.94 × 10^–6^ J/cm^2^ for PVP/MnO_2_-12 and PVP/MnO_2_-15 composite nanofibers, respectively. The lower onset optical limiting threshold was obtained for the sample with the highest MnO_2_ nanofiller content. This was due to the stronger NA behavior of the PVP/MnO_2_-15 nanofibers. Compared to other nonlinear optical materials at 532 nm, such as benchmark optical limiting material *C*_60_ (3.0 J/cm ^2^), graphene oxide nanosheets (>3.0 J/cm^2^), carbon nanotubes (1.4 J/cm^2^), and MoS_2_ nanotubules (1.1 J/cm^2^),^77−79^ it can be clearly seen that the present composite nanofibers showed the best optical limiting performance with the lowest limiting threshold values. Considering all of the obtained results, the PVP/MnO_2_ composite nanofibers have excellent optical limiting performance.

## 4 Conclusions

In this study, MnO_2_ nanoparticles were synthesized by the reduction method, and PVP/MnO_2_ composite nanofibers were produced by electrospinning to enhance the light-matter interaction-related NA behavior-triggered optical limiting features of PVP nanofibers. SEM images of the composite nanofibers show that MnO_2_ nanoparticles were well incorporated into the nanofibers. Linear optical measurements indicate increased band gap values, from 3.95 to 4.07 eV, and Urbach energy, from 1.46 to 1.71 eV, with increasing MnO_2_ nanofiller content. Besides, the photoluminescence measurement results indicate extended localized defect states at around 2.29 eV, which are below the conduction band within the band gap. To enhance the NA behavior using the localized defect states, the energy of the excitation wavelength was chosen at 532 nm (2.32 eV) in OA Z-scan experiments. All of the composite nanofibers show NA behavior, which becomes weaker as the input intensity is increased due to the filling of the defect states by OPA. Considering the localized defect states and band gap energies of both composite nanofibers, their possible NA mechanisms are OPA, sequential/simultaneous TPA, and ESA. The higher β_eff_ value was obtained for the sample with the highest MnO_2_ nanofiller content (PVP/MnO_2_-15) at 1.18 × 10^–5^ m/W at lower input intensity. This was attributed to the higher amount of localized defect states, which causes stronger NA. The lower onset optical limiting threshold was found to be 2.94 × 10^–5^ J/cm^2^ for PVP/MnO_2_-15 composite nanofibers. Their robust NA behavior, associated with their strong optical limiting properties, renders them highly suitable candidates for efficient optical limiting applications.
